# Crop rotation increases Tibetan barley yield and soil quality on the Tibetan Plateau

**DOI:** 10.1038/s43016-024-01094-8

**Published:** 2025-01-28

**Authors:** Hui Wu, Enke Liu, Tao Jin, Buchun Liu, Subramaniam Gopalakrishnan, Jie Zhou, Guodong Shao, Xurong Mei, Pierre Delaplace, Caroline De Clerck

**Affiliations:** 1 Institute of Environment and Sustainable Development in Agriculture, Chinese Academy of Agricultural Sciences, Beijing, China; 2 Plant Sciences, Gembloux Agro-Bio Tech, Liege University, Gembloux, Belgium; 3 State Key Laboratory of Efficient Utilization of Agricultural Water Resources, CAU/CAAS, Beijing, China; 4 Tibetan Academy of Agricultural and Animal Husbandry Sciences, Lhasa, China; 5 State Key Laboratory of Hulless Barley and Yak Germplasm Resources and Genetic Improvement, Lhasa, China; 6 International Institute of Tropical Agriculture, Dar es Salaam, Tanzania; 7 College of Agriculture, Nanjing Agricultural University, Nanjing, China; 8 Geo-Biosphere Interactions, Department of Geosciences, University of Tübingen, Tübingen, Germany

**Keywords:** Plant ecology, Agroecology, Agroecology

## Abstract

Tibetan barley (*Hordeum vulgare*) accounts for over 70% of the total food production in the Tibetan Plateau. However, continuous cropping of Tibetan barley causes soil degradation, reduces soil quality and causes yield decline. Here we explore the benefits of crop rotation with wheat and rape to improve crop yield and soil quality. We conducted 39 field experiments on the Tibetan Plateau, comparing short-term (≤5 years), 5–10 years and long-term (≥10 years) continuous cropping with rotation of Tibetan barley with wheat or rape. Results showed that Tibetan barley–wheat and Tibetan barley–rape rotations increased yields by 17% and 12%, respectively, while improving the soil quality index by 11% and 21%, compared with long-term continuous cropping. Both Tibetan barley rotations with wheat and rape improved soil quality and consequently yield, mainly by increasing soil microbial biomass nitrogen and microbial biomass carbon and decreasing pH. By contrast, long-term continuous cropping led to decreased soil organic matter, lower microbial biomass nitrogen and increased pH, contributing to yield decline. The benefits of rotations on crop yield and soil quality increased over time. Implementing crop rotation with wheat or rape thus offers a sustainable agricultural strategy for improving food security on the Tibetan Plateau.

## Abstract (web summary)

Continuous cropping of Tibetan barley results in rapid declines in both yield and soil quality due to the low baseline soil fertility in the Tibetan Plateau. This study, based on 39 field experiments, showed that rotating Tibetan barley with wheat or rape increased yields and improved the soil quality compared with long-term continuous cropping.

## Main

The Tibetan Plateau provides water for about 40% of the global population^1^. Its ecological stability is crucial for the sustainable development of agriculture and animal husbandry in Asia. As the highest plateau in the world, the Tibetan Plateau faces challenges for crop growth due to its low average annual temperature and low oxygen levels. Soil degradation in the Tibetan Plateau region under the influence of multiple factors is a serious threat to soil erosion and food security^2,3^. One of the crucial drivers of this impending problem is soil degradation caused by conventional agriculture^4,5^. Among conventional agriculture practices, continuous cropping (continuous monoculture) commonly leads to nutrient imbalance and land degradation, which limit soil quality and cause environmental damage^6^ negatively affecting key functions of the soil. Continuous monoculture of wheat and green bean will cause soil degradation thereby reducing crop yield^7,8^. The response of soil quality and crop yield to continuous cropping was different for different crops and regions^6^. In southern Denmark, 36 years of continuous barley cropping reduced yields by about 50%^9^. The results of a 6 year Tibetan barley cropping experiment on the Tibetan Plateau showed a 54% and 36% reduction in soil available nitrogen and yield, respectively^10,11^. Currently, the main research direction of Tibetan barley continuous cropping (CC) on the Tibetan Plateau focuses on the changes of certain elements in individual experiments and lacks comprehensive evaluation of their soil quality and yield.

Crop rotations can increase environmental sustainability, agroecosystem diversity and soil quality^12–15^. Four-year rotation (spring wheat–turnip rape–barley–pea) improved soil fertility and crop yield and reduced wheat diseases compared with wheat monocultures^16^. In India, 29 years of continuous wheat crop significantly reduced crop yield, soil organic matter (SOM), total nitrogen (TN) contents and earthworm population compared with wheat–rice rotation^17^. The barley–vetch rotation increased yields by 45% over the barley continuous cropping after 4 years^18^. Currently, crop rotation on the Tibetan Plateau mainly focuses on pasture research for animal husbandry, with large gaps in agriculture. To improve the ecological stability of the Tibetan Plateau, a comprehensive study of the effects of crop rotation of major food crops on soil quality and yield on the Tibetan Plateau is necessary. The soil quality index (SQI) provides a simple metric that evaluates the overall function of soil by aggregating multiple variables into a single value^19^, allowing for consideration of trade-offs between ecosystem functions^20^.

Tibetan barley has become the major food crop on the Tibetan Plateau, accounting for more than half of the total crop yield^21–23^. With unsuitable climatic conditions and few crop options available, continuous Tibetan barley cultivation has become an emergent problem in the Tibetan Plateau region. Wheat and rape are the second and third common crops on the Tibetan Plateau. Therefore, we studied the changes in crop yield and soil quality of fields cultivated with Tibetan barley continuously in short term (for less than or equal to 5 years), 5–10 years and long term (for 10 years), as well as cropping rotation with wheat and rape in alternation for the corresponding years. The objectives of this study were to explore the effects of different types and durations of Tibetan barley rotations on soil quality and yield on the Tibetan Plateau, identify the key factors influencing crop yield and soil quality in these rotations, and determine the most suitable rotation planting pattern for Tibetan barley in this region.

## Results

### Effects of Tibetan barley rotation on yield

**Fig. 1:**
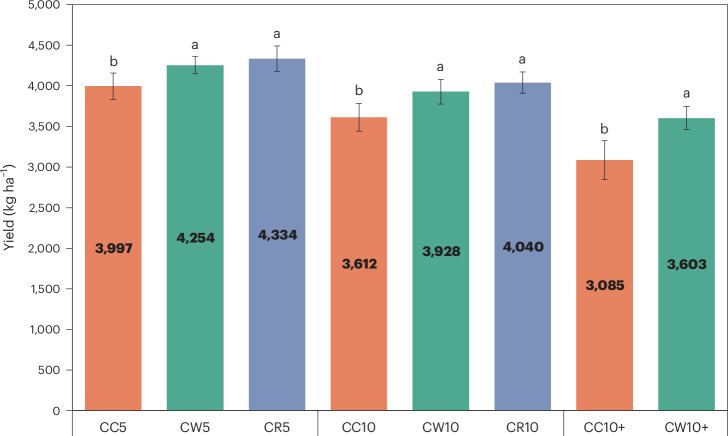
Average yield of Tibetan barley with long-term continuous and rotational cropping. Orange for CC, green for CW, purple for CR. *n* = 8, *n* = 5, *n* = 6, *n* = 5, *n* = 3, *n* = 3, *n* = 6, *n* = 3 in CC5, CW5, CR5, CC10, CW10, CR10, CC10+, CW10+, respectively. Biological replicates were used for each treatment. Data are presented as mean ± standard deviation. Different letters indicate significant differences among Tibetan barley yields as calculated by Duncan multiple comparative analysis. No correction for multiple testing was applied. The results of Duncan multiple comparative analysis are shown in Supplementary Table 6. Source data

A total of 39 Tibetan barley fields were selected in the Tibetan Plateau regions for soil chemical properties testing and yield statistics (Supplementary Table 1). The yield of CC10 (5–10 years of CC) and CC10+ (≥10 years of CC) decreased by 10% and 23%, respectively, compared with CC5 (≤5 years of CC) (Fig. 1 and Supplementary Table 1). The yields of CW5 (≤5 years of Tibetan barley–wheat rotation (CW)), CW10 (5–10 years of CW) and CW10+ (≥10 years of CW) were 6%, 9% and 17% higher than those of CC5, CC10 and CC10+, respectively. The yield of CR5 (≤5 years of Tibetan barley–rape rotation (CR)) and CR10 (5–10 years of CR) were 8% and 12% higher than that of CC5 and CC10, respectively.

### Effects of continuous cropping on Tibetan barley soil

**Extended Data Fig. 1:**
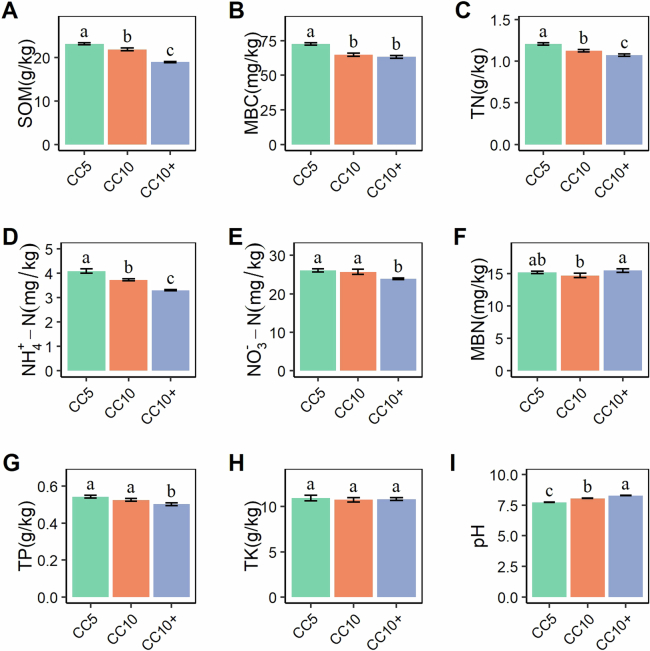
SOM, MBC, TN, NH_4_^+^-N, NO_3_^−^-N, MBN, TP, TK and pH in different lengths of continuous Tibetan barley crop. (**a**) SOM, soil organic matter. (**b**) MBC, microbial biomass carbon. (**c**) TN, total nitrogen. (**d**) NH_4_^+^-N. (**e**) NO_3_^−^-N. (**f**) MBN, microbial biomass nitrogen. (**g**) TP, total phosphorus. (**h**) TK, total potassium. (**i**) pH. <5. CC5, CC10, and CC10+ represent Tibetan barley continuous cropping treatments for less than or equal to 5, 5–10 and more than or equal to 10 years, respectively. n = 24, n = 15, n = 18 in CC5, CC10, CC10+, respectively. Biological replicates were used for each treatment. Data are presented as mean ± standard error. Different letters indicated significant differences among Tibetan barley yields as calculated by Duncan multiple comparative analysis, respectively. No correction for multiple testing was applied. The results of Duncan multiple comparative analysis are shown in Supplementary Table 6.

SOM and microbial biomass carbon (MBC) were examined to investigate the effect of different crop years on the soil carbon content of Tibetan barley fields. The content of SOM in Tibetan barley significantly decreased with increasing years of CC: 23.12 g kg^−1^, 21.83 g kg^−1^ and 18.94 g kg^−1^ in CC5, CC10 and CC10+, respectively (Extended Data Fig. 1a and Supplementary Table 2). Soil MBC of Tibetan barley fields CC5, CC10 and CC10+ were 72.65 mg kg^−1^, 64.83 mg kg^−1^ and 63.28 mg kg^−1^, respectively (Extended Data Fig. 1b). Soil MBC content was significantly different between CC5 and CC10. However, there were no significant differences between CC10 and CC10+. The highest microbial biomass nitrogen (MBN) content of 15.47 mg kg^−1^ in Tibetan barley field soil was obtained for CC10+ and the lowest MBN content of 14.71 mg kg^−1^ for CC10 (Extended Data Fig. 1f).

The soil TN content was significantly reduced by continuous Tibetan barley cropping (Extended Data Fig. 1c). Soil TN content of CC5 was 1.21 g kg^−1^ and was reduced by 7% and 11% for CC10 and CC10+, respectively. Ammoniacal nitrogen (NH_4_^+^-N) content of soils planted with Tibetan barley in CC5, CC10 and CC10+ was 4.08 mg kg^−1^, 3.72 mg kg^−1^ and 3.29 mg kg^−1^, respectively (Extended Data Fig. 1d). Nitrate nitrogen (NO_3_^−^-N) content of soils planted with Tibetan barley in CC5, CC10 and CC10+ was 26.08 mg kg^−1^, 25.73 mg kg^−1^ and 23.93 mg kg^−1^, respectively (Extended Data Fig. 1e). Soil NH_4_^+^-N and NO_3_^−^-N contents were significantly different from CC10+ in CC10.

The soil total phosphorus (TP) content decreased as the number of years of continuous Tibetan barley crop increased (Extended Data Fig. 1g). Soil TP content was 0.53 g kg^−1^ for CC10 and was not significantly different from that for CC5. Soil total potassium (TK) content in Tibetan barley fields was least at 10.75 g kg^−1^ in CC10 (Extended Data Fig. 1h). The impact of continuous cropping of Tibetan barley on soil TK was not significant. pH significantly increased with increasing years of continuous Tibetan barley crop (Extended Data Fig. 1i).

### Differences in soil between continuous cropping and rotation

**Fig. 2:**
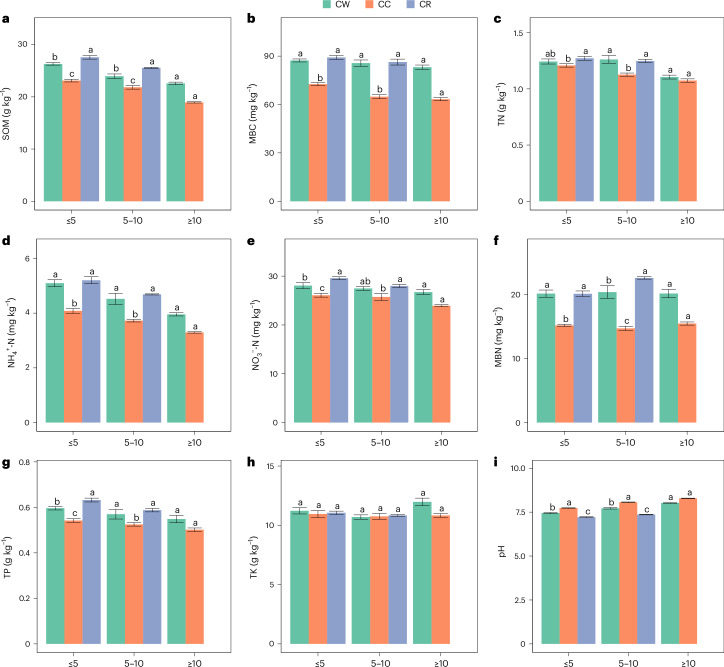
Differences in SOM, MBC, TN, NH_4_^+^-N, NO_3_^−^-N, MBN, TP, TK and pH in different years of continuous and rotation Tibetan barley crop. **a**, SOM. **b**, MBC. **c**, TN. **d**, NH_4_^+^-N. **e**, NO_3_^−^-N. **f**, MBN. **g**, TP. **h**, TK. **i**, pH. CC treatments: ≤5, less than or equal to 5 years; 5–10, 5–10 years; ≥10, more than or equal to 10 years. *n* = 24, *n* = 15, *n* = 18, *n* = 15, *n* = 9, *n* = 9, *n* = 18, *n* = 9 in CC5, CW5, CR5, CC10, CW10, CR10, CC10+, CW10+, respectively. Biological replicates were used for each treatment. Data are presented as mean ± standard error. Different letters indicate significant differences among Tibetan barley yields as calculated by Duncan multiple comparative analysis. No correction for multiple testing was applied. The results of Duncan multiple comparative analysis are shown in Supplementary Table 6. Source data

Long-term Tibetan barley continuous crop and crop rotation both significantly reduced the SOM content compared with short-term cropping (Fig. 2a and Supplementary Table 2). CW and CR significantly increased SOM content compared with CC. The SOM content of all three cropping patterns decreased with the increase of cropping years. The soil MBC content in the rotations was significantly higher than that in continuous cropping (Fig. 2b). Soil MBC content of CR5 and CR10 was 23% and 33% higher than that of CC5 and CC10, respectively. The soil MBC content of the two crop rotation patterns decreased with the number of years of cropping.

Tibetan barley rotation with rape increased the soil TN content in all three rotation years compared with continuous cropping (Fig. 2c). CW10 significantly increased the soil TN content compared with CC10. Long-term CC and rotation significantly reduced soil NH_4_^+^-N contents (Fig. 2d). CW and CR significantly increased soil NH_4_^+^-N content. Soil NH_4_^+^-N content of CW5 and CR5 was 25% and 27% higher than that of CC5, respectively. CC5 and CW5 had significantly lower soil NO_3_^−^-N content than CR5, and CC10 had significantly lower soil NO_3_^−^-N content than CR10 (Fig. 2e). Soil MBN content of CR10 was 45% higher than that of CC10 (Fig. 2f).

The TP content of CW and CR was significantly higher than that of CC (Fig. 2g). The TP content of CR5 was significantly higher than that of CC5 and CW5, with an increase of 17% and 5%, respectively. There was no significant difference in soil TK content with the cropping patterns when the cultivation time was less than 10 years (Fig. 2h). The pH of CC was significantly higher than those of CW and CR (Fig. 2i). Soil pH increased with increasing years of continuous and rotation Tibetan barley crop.

**Extended Data Fig. 2:**
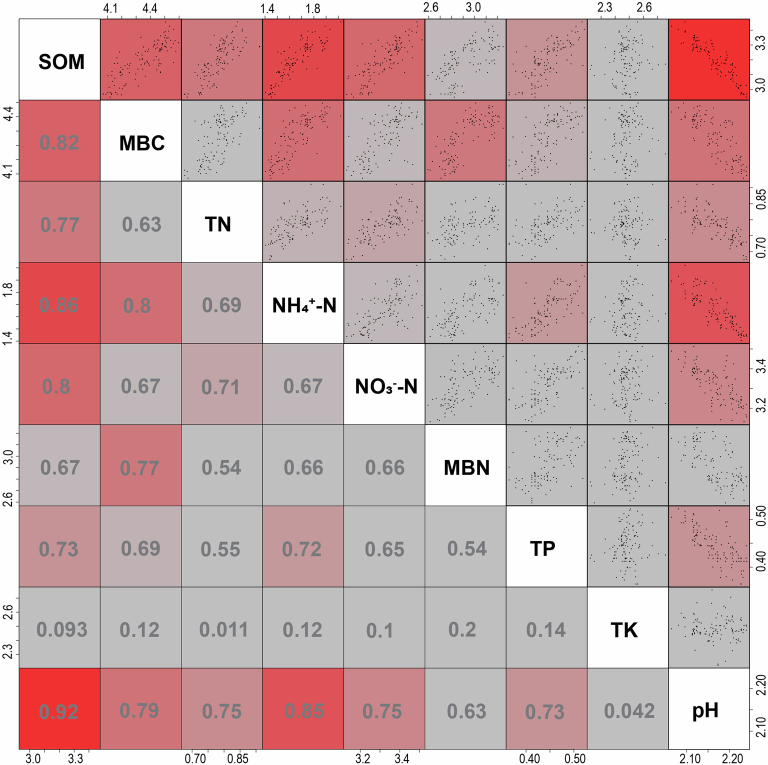
Correlation analysis of soil properties in Tibetan barley fields. The module in the lower left corner represents the magnitude of the correlation between the two soil properties, and the module in the upper right corner represents the trend distribution of the correlation between the two soil properties. The redder the color of the module, the higher the correlation. Source data

The correlation analysis of nine soil properties in the fields of Tibetan barley shows that the correlation between SOM and pH is the highest, and it is negatively related (Extended Data Fig. 2 and Supplementary Table 3). The changes in TK content of Tibetan barley fields showed no significant correlation with other soil properties, and its correlation with TN was the weakest of all. The changes in soil carbon and nitrogen content in the field of Tibetan barley were significantly positively correlated with the changes in MBC and MBN content.

### SQI for continuous cropping and rotation

**Extended Data Fig. 3:**
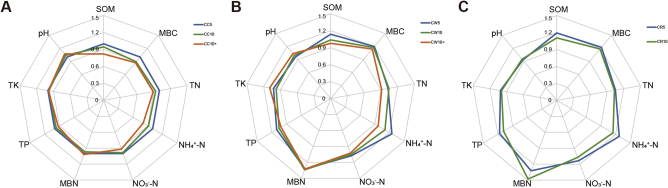
The SQI of Tibetan barley soil in three cropping patterns in different cropping years. (**a**) Tibetan barley continuous crop. (**b**) Tibetan barley-wheat rotation. (**c**) Tibetan barley-rape rotation. The SQI of Tibetan barley soil under three cropping patterns in the same cropping year. And the SQI of Tibetan barley soil under three cropping patterns in the same cropping years. Blue for less than or equal to 5 years, green for 5–10 years, orange for more than or equal to 10 years.

SQI can be used to measure the degree of soil degradation and soil health^19^. We analysed SQI for different years of CC and rotation. SQI showed a decreasing trend with increasing years of continuous Tibetan barley crop, and the decreasing trend of Tibetan barley continuous crop for CC5 years and CC10+ years was higher than for CC10 (Extended Data Fig. 3a and Supplementary Table 4). The SQIs of CC5, CC10 and CC10+ were 1.00, 0.93 and 0.87, respectively. The SQI of long-term rotation of Tibetan barley with wheat and rape was significantly lower than that of short-term rotation (Extended Data Fig. 3b,c).

**Fig. 3:**
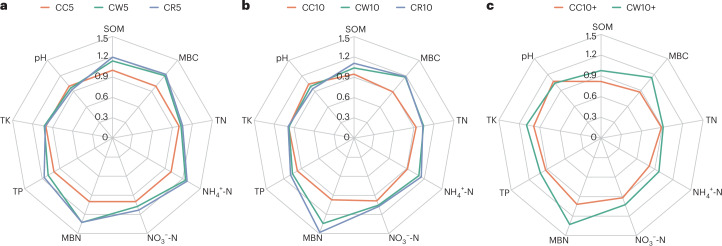
The SQI of Tibetan barley soil under three cropping patterns in the same cropping year. **a**, Less than or equal to 5 years. **b**, 5 to 10 years. **c**, More than or equal to 10 years. Source data

**Extended Data Fig. 4:**
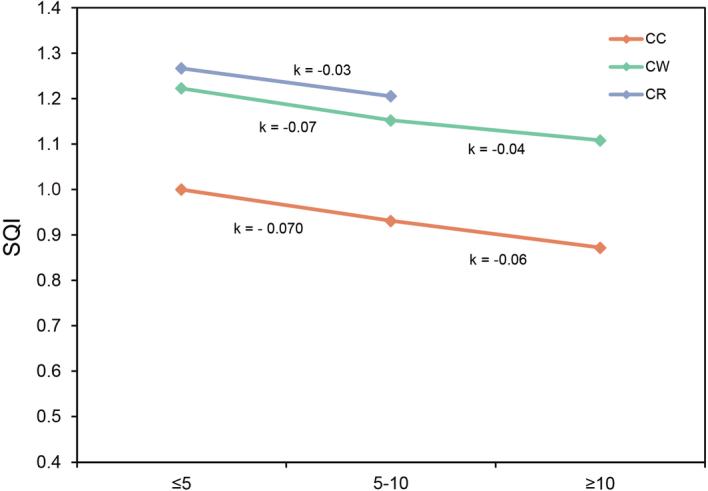
Variation trend of SQI of Tibetan barley continuous cropping and rotation cropping. ≤5, 5-10, and ≥10 represent Tibetan barley continuous cropping treatments for less than or equal to 5, 5-10 and more than or equal to 10 years, respectively. The results of SQI are shown in Supplementary Table 4.

When considering the same cropping year, the SQI of continuous cropping of Tibetan barley was the lowest among the three planting patterns in all three planting periods. The SQI of CR5 and CR10 was higher than CW5 and CW10 (Fig. 3a,b). The SQI of CW10+ was significantly higher than that of CC10+ (Fig. 3c). The ratio of SQI area in CW10+ and CR10 to area in CC5 were 1.15 and 1.21. SQI of the range from CC5 to CC10 dropped faster than that of the range from CC10 to CC10+ (Extended Data Fig. 4). The SQI of CC dropped faster than that of CW and CR.

### Key factors for changes in soil properties

**Fig. 4:**
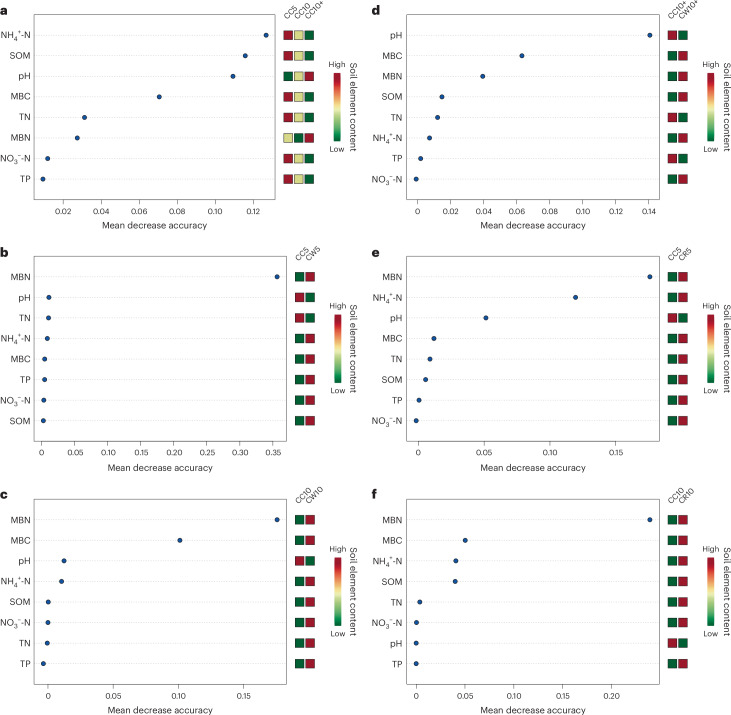
Machine learning of soil characteristics of continuous and rotational Tibetan barley crops. **a**, Tibetan barley continuous crop. **b**, Comparison of continuous and rotation with wheat in Tibetan barley for less than or equal to 5 years. **c**, Comparison of continuous and rotation with rape in Tibetan barley for less than or equal to 5 years. **d**, Comparison of continuous and rotation with wheat in Tibetan barley for 5 to 10 years. **e**, Comparison of continuous and rotation with rape in Tibetan barley for 5 to 10 years. **f**, Comparison of continuous and rotation with wheat in Tibetan barley for more than or equal to 10 years.

The key factors for long-term continuous cropping were changes in soil chemical properties in Tibetan barley, including NH_4_^+^-N, SOM, pH and MBC (Fig. 4a). Soil MBN and pH had a key role in the difference between CC5 and CW5 (Fig. 4b). Soil MBN, NH_4_^+^-N, pH and MBC had also a key role in the difference between CC5 and CR5 (Fig. 4e). The main factors for soil differences in both CW10 and CR10 compared with CC10 were MBN and MBC (Fig. 4c,f). The main factors influencing soil differences between CW10+ and CC10+ were soil pH and MBC contents (Fig. 4d).

### Key factors for changes in SQI and yield

**Fig. 5:**
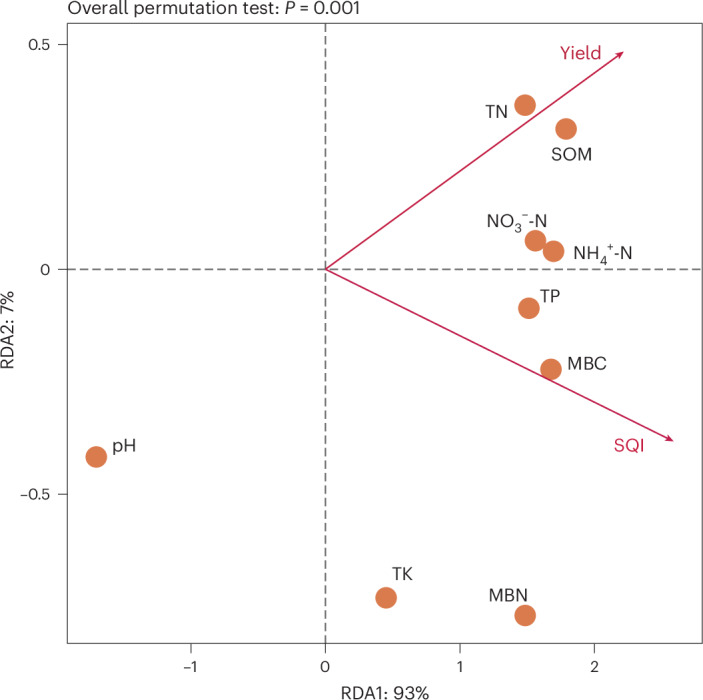
Redundancy analysis of Tibetan barley yield and SQI to soil characteristics in long-term continuous and rotational crops. Scatterplots show the first (RDA1) versus the second (RDA2) dimension. The statistical test used was two-sided.

**Extended Data Fig. 5:**
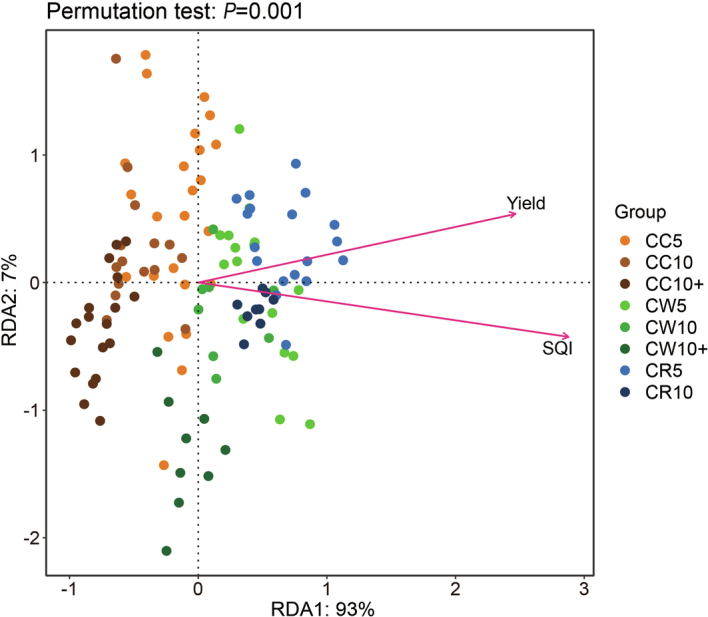
Correlation of Tibetan barley yield and SQI with cropping pattern and cropping years. The statistical test used was two-sided.

Redundancy analysis was used to study the correlation between Tibetan barley yield and SQI with different soil conditions at different years of cropping patterns. Tibetan barley yield had a positive correlation with SQI (Fig. 5). Tibetan barley SQI had a significant positive correlation with soil MBC, MBN, NO_3_^−^-N and TP. Tibetan barley yield had a significant positive correlation with soil SOM and TN. Tibetan barley yield and SQI were negatively correlated with pH. In terms of Tibetan barley cropping patterns, yield and SQI were significantly negatively correlated with continuous Tibetan barley cropping (Extended Data Fig. 5).

**Fig. 6:**
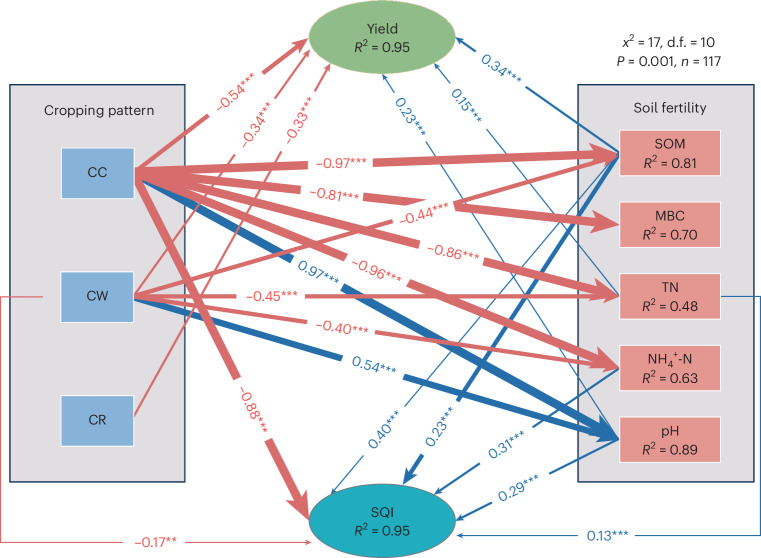
SEM of crop rotation effects on soil properties and consequent yield. ***P < 0.001; **P < 0.01. Blue and red arrows indicate positive and negative relationships between variables, respectively. The arrow width is proportional to the strength of the relationship. The proportion of variance explained is given by each response variable (*R*^2^), and measures of overall model fit are shown in the upper right-hand corner. Non-significant paths are not shown. Full model results are given in Supplementary Table 5. *x*^2^, chi-square; d.f., degrees of freedom; *n*, sample size. Source data

Structural equation modelling (SEM) was further used to evaluate the direct and indirect effects of soil properties on soil quality and yield under Tibetan barley rotation (Fig. 6 and Supplementary Table 5). Long-term continuous cropping and rotation both reduced Tibetan barley yield and SQI, but CR was significantly lower than CW, which was significantly lower than CC. The main reasons of reduced Tibetan barley yield and SQI in long-term continuous cropping include SOM, MBC, TN and NH_4_^+^-N. The main causes of reduced Tibetan barley yield and SQI in long-term wheat rotation include SOM, TN and NH_4_^+^-N. CW and CR mainly improved the Tibetan barley yield and SQI by reducing soil pH and increasing soil quality. In addition, SOM was a key factor affecting yield and SQI in Tibetan barley fields.

## Discussion

### Effect of continuous cropping on soil quality and yield

Tibetan barley yield and soil quality showed a significant decreasing trend with increasing years of continuous cropping. Soil pH of Tibetan barley field showed a significant negative correlation with Tibetan barley yield and soil quality. Freeze–thaw action in Tibetan Plateau soils may promote soil alkalization^24^. In addition, there is a potential for alkalization of soils under continuous cropping of *Poaceae* crops due to root secretions^25^. The regulating effect of organic acids produced by decomposition of SOM on soil pH may be the reason for the significant negative correlation between SOM and pH content in CC soils^26^. SOM is proportional to the soil’s total content in organic carbon and is the main source of soil nutrients^27–29^. SOM can affect soil physical properties, soil structure and water retention capacity^30,31^. The level of SOM content plays an important role in the yield and quality of crops^32,33^. Humus can effectively adsorb soil pollutants to alleviate soil pollution^34^. In recent decades, soil carbon content in the Tibetan Plateau has hardly changed^35^. Therefore, long-term continuous cropping is harmful to the sustainability of the soil^36^. SQI, machine learning redundancy and SEM analysis all showed that long-term continuous cropping negatively impacted Tibetan barley yield and soil quality, in which reduced SOM plays a key role. Soil TP content and SOM content showed an identical trend. The result of machine learning showed that SOM was more important for the degradation of continuous Tibetan barley field soil. Meta-analysis showed that the addition of phosphorus in farmland increased the soil carbon content^37^. Soil carbon and phosphorus utilization in Tibetan barley fields might have a coupling effect^38^.

Soil TN, NH_4_^+^-N and NO_3_^−^-N contents significantly reduced due to continuous Tibetan barley culture. The result of machine learning showed that declining soil NH_4_^+^-N content was the most important factor in the degradation of soil in continuous cropping in Tibetan barley fields. Soil NH_4_^+^-N is a form that can be used directly by crops, and reduced soil NH_4_^+^-N levels seriously affect crop nitrogen^39^. Redundancy analysis showed that the correlation between soil NH_4_^+^-N and yield was greater than that between NO_3_^−^-N and yield. SEM results showed that the impact of continuous cropping on soil NH_4_^+^-N was greater than that on TN. Continuous cropping of Tibetan barley caused an increase in pH, which may be related to the accumulation of root exudates from Tibetan barley. This accumulation of soil alkalinity could also affect soil quality and yield.

Both soil MBC and MBN tended to decrease with increasing years of CC. Soil MBC and MBN contents were the lowest at CC10+. A meta-analysis showed that soil MBC and MBN increased by 14.3% and 20.1%, respectively, over the Tibetan Plateau with climate warming in recent decades^40^. In fact, the contents of soil MBC and MBN in Tibetan barley with long-term continuous cropping showed a larger decreasing trend. Long-term continuous cropping of Tibetan barley reduced soil microbial biomass. Soil MBC content showed the highest positive correlation with SQI of Tibetan barley fields. The reduction of soil MBC and MBN was detrimental to the material cycle of Tibetan barley fields and had a negative impact on Tibetan barley growth.

The continuous cropping of Tibetan barley has resulted in the production of specific rhizosphere secretions and the absorption of soil nutrients, thereby deteriorating soil quality, which is characterized by increased soil pH and decreased soil NH_4_^+^-N, SOM and MBC content. This deterioration of soil quality under continuous cropping of Tibetan barley negatively impacts the physiological functions (such as carbon fixation) and growth (such as yield) of Tibetan barley. The negative feedback regulation between Tibetan barley growth and soil quality exacerbates the decline in Tibetan barley yield and soil quality as the years of continuous cropping increase. Notably, the decline in soil quality becomes severe within the first 5 years of continuous cropping of Tibetan barley. Even for short-term continuous cropping, the practice of continuous cropping of Tibetan barley should be minimized.

### Effect of cropping rotation on soil quality and crop yield

CW and CR significantly improved Tibetan barley yield and soil quality in the short to long term compared with continuous barley crops. The decline in SOM content and soil alkalization were the key factors leading to the decline in yield and soil quality in the CC. SOM content of CR was significantly higher than that of CW which was significantly higher than that of barley continuous cropping. However, soil pH showed an opposite trend. Correlation analyses showed that SOM in the Tibetan barley field had the highest correlation with pH. SEM analyses showed that pH and SOM had the greatest effect on SQI and Tibetan barley yield. Continuous cropping of Tibetan barley resulted in higher soil pH and lower SOM, which in turn led to lower soil quality. CR significantly mitigated this phenomenon. Rape rotations had the potential to increase SOM and mitigate pH alkalization^41,42^. The high-quality carbon sequestration capacity of wheat might be the reason for the increase of SOM in the soil of CW^43^. Tibetan barley rotation with wheat was not as effective as rotation with rape in mitigating the pH of continuously cropped Tibetan barley fields, which may be due to the fact that both wheat and Tibetan barley belong to the *Poaceae* family. The SQI of CC dropped faster than that of CW and CR in the past. Due to the increase in pH with the continuous cropping of Tibetan barley, the SQI of continuous cropping of Tibetan barley had decreased faster than what is shown in Extended Data Fig. 2. The decrease in SQI could be mitigated more effectively by crop rotation. Both of two crop rotation patterns can be used to ameliorate the problem of declining soil quality and Tibetan barley yield load caused by continuous Tibetan barley crop.

Tibetan barley rotation with both wheat and rape could significantly ameliorate the problem of decreasing soil TP content with long-term continuous cropping. The effect of short-term Tibetan barley rotation on soil TK was not significant, but long-term rotation increased TK content. Crop rotation is an effective method of increasing soil TN, even more than N fertilizer inputs^44,45^. This might be the reason why soil TN was significantly higher in Tibetan barley rotation with both wheat and rape soils than in continuous cropping despite the presence of urea applied for 5–10 years. Although Tibetan barley rotations mitigated the rate of decline in soil SOM and TN content, long-term rotations of Tibetan barley also had a declining trend. No-planting measures could be considered to restore soil quality in arable fields with low soil quality that have been continuously planted for decades. SEM showed that the increase of MBC and MBN NH_4_^+^-N in CW and CR was the important reason for the increase of Tibetan barley yield and SQI. The well-developed root system of rape has the ability to improve the soil structure of Tibetan barley fields^41,46^. Changes in soil structure affected microecology and material cycling in Tibetan barley fields. Wheat and rape diversity root secretions had the potential to increase soil microbial diversity. In terms of soil microbial carbon and nitrogen, CW and CR were high-quality cropping patterns for alleviating the continuous crop obstacles.

Currently, research on agriculture and animal husbandry on the Tibetan Plateau have mainly focused on animal husbandry and climate warming-induced impacts. Despite its fragile agroecosystems, low crop selectivity and low productivity, the Tibetan Plateau has a large area of cultivated land. Among them, Tibetan barley is the main food crop in the Tibetan Plateau, accounting for more than 70% of the total food crop area. The sustainable development of Tibetan barley cultivation contributes to the stability of the Tibetan Plateau in the face of global climate change. Due to the low baseline soil fertility in the Tibetan Plateau, the continuous cropping of Tibetan barley leads to a rapid decline in yield and soil quality. However, crop rotation can significantly mitigate this downward trend. Next, exploring suitable field management methods for Tibetan barley rotation can further tap the sustainable development potential of Tibetan barley production in the Tibetan Plateau.

## Methods

### Site description and sample evaluation

In March 2022, a systematic survey of Tibetan barley fields was carried out in the main production area of Tibetan barley in the Tibetan valley agricultural region. The Tibetan valley agricultural region has a plateau temperate semi-arid monsoon climate. The average annual temperature at all experimental sites is between 6 °C and 8 °C, and the average annual precipitation is between 450 mm and 550 mm. In the valley agricultural region of the Tibetan Plateau, 39 fields of Tibetan barley were selected, including CC, CW, CR (Supplementary Table 1). Thirty-nine experimental plots in different cropping years were co-managed by the Tibet Academy of Agricultural and Animal Husbandry Sciences and farmers for crop rotation and continuous cropping research. The 39 plots of Tibetan barley fields were all larger than 2,000 m^2^, with the soil type being sandy loam. All the plots were planted with Tibetan barley in the same year, with the same variety ‘Zangqing 2000’. All the fertilization methods involved the application of basal fertilizer before planting, including 150–200 kg hm^−^^2^ of urea (N 46%), 100–150 kg hm^−^^2^ of superphosphate (including P_2_0_5_ 16%) and 50–100 kg hm^−^^2^ of potassium chloride (KCl 60%). In addition, to mitigate the differences in altitude, oxygen levels, soil types and other factors, the experimental fields of the three planting methods selected were evenly distributed in the valley regions of the Tibetan Plateau. We chose CC and rotation fields that had as similar growth conditions as possible, same environments and planting methods. To further alleviate environmental differences, we divided the planting years into categories as follows: less than or equal to 5 years, 5 to 10 years and greater than or equal to 10 years for subsequent analysis. CC5, CC10 and CC10+, as well as the CW5, CW10 and CW10+, and CR5 and CR10 were selected for this study.

Soil sampling in these fields was conducted before Tibetan barley planting, from 5 April to 15 April 2022. Three surface composite soil samples (0–20 cm) (made of 5 cores) were taken along the diagonal of each Tibetan barley field. Therefore, we collected a total of 126 samples. Soils were homogenized and sieved through 2 mm, and visible shoot litter and root, as well as stones, were picked out. Then each sample was separated into two parts. One was stored at 4 °C and was used to measure soil MBC and MBN within 1 week. The other part was air-dried for the determination of soil physicochemical properties. In September 2022, the yield data of Tibetan barley at the sampling points were collected from the farm yield record table (Fig. 1 and Supplementary Table 1).

### Determination of soil physiochemical properties

SOM was determined using the potassium dichromate volumetric method^47^. The modified Kjeldahl method was used for the determination of soil TN^48^. The samples were analysed for NH_4_^+^-N and NO_3_^−^-N using a continuous flow analyser^49^. The molybdenum blue colorimetric method was used for the analysis of soil TP^50^. The flame photometric method was used for the determination of TK^51^. Soil pH was assayed by a pH meter in soil water suspensions (1:2.5 wt/volume)^52^. The chloroform fumigation method was adopted to determine soil MBC and MBN using a total organic carbon analyser (multi N/C 3100)^53^.

### SQI and SEM

For the SQI analysis of Tibetan barley fields, nine soil characteristics were considered, including SOM, TN, NH_4_^+^-N, NO_3_^−^-N, TP, TK, pH, MBC and MBN. First, the CC5 soil quality was set to a regular nine-sided polygon of radius 1 based on the soil’s physiochemical properties. Next, the radius of the other soil quality nine-sided polygon was the ratio of the soil physiochemical properties data divided by the CC5 data^19^. The nine radius lengths of the nine-sided shape were determined based on the ratio of the content of each treatment to the content of the nine soil characteristics of the CC5. The ratio of each treatment’s nine-sided area to the CC5 nine-sided area was the SQI for that treatment. The SQI value for the CC5 soil was 1. The ratio of the different patterns to the CC5 nine-sided polygon area was the soil’s SQI of the Tibetan barley fields. Lastly, visualization was carried out using Excel 2019.

Lavaan, haven, Hmisc and semPlot packages in R4.3.2 software were used for SEM analysis. The SEM fitness was examined on the basis of a non-significant chi-square test (*P* > 0.05), the goodness-of-fit index, the degree of freedom and the root mean square error of approximation. CC, CW and CR soil properties (including soil SOM, TN, NH_4_^+^-N, NO_3_^−^-N, TP, TK, pH, MBC, MBN, SQI) and yield data were used for SEM construction. SEM was constructed by quantitatively categorizing the different planting years of CC, CW and CR, where less than or equal to 5 years was designated as 1, 5–10 years was quantified as 2, and more than or equal to 10 years was quantified as 3. Finally, the optimal SEM construction was completed using soil TN, NO_3_^−^-N, MBN, TK, pH, SQI and yield of continuous and rotation cropping of Tibetan barley.

### Statistical analysis

To balance the variability of sample growing conditions, we divided the serial sample treatments into less than or equal to 5 years, 5 to 10 years and more than or equal to 10 years after the completion of soil quality testing for statistical analysis. One-way analysis of variance test and Duncan test were applied to test the yield differences among treatments at *P* < 0.05 using the SPSS 26.0. Excel_2021 was used for data visualization of yield. A correlation analysis of the nine chemical properties within the same group was conducted in the barley field using the corrgram package in R language. Covariance and correlation coefficients were used to determine and visualize the intra-group correlation of nine soil properties in Tibetan barley fields. Analysis of variance test and Duncan test of differences among treatments at *P* < 0.05 in soil chemical properties were performed using the agricolae package in R4.3.2 software^54^ and then visualized using ggplot2^55^. Excel_2021 was used to create radar plots and analyse the SQI. The R language’s randomForest package was used to perform a random forest importance analysis on the nine soil properties between treatments and determine the order of importance of soil properties between treatments. Finally, using R language’s vegan, ggrepel, ggplot2 and ggpubr packages, we conducted a redundancy analysis with SQI and yield as the dependent variables and the nine soil properties as the independent variables.

### Reporting summary

Further information on research design is available in the Nature Portfolio Reporting Summary linked to this article.

## Supplementary information

Supplementary Information Supplementary Tables 1–6, Fig. 2 code, Fig. 4 code, Fig. 5 code, Fig. 6 code, Extended Data Fig. 1 code and Extended Data Fig. 2 code.

Reporting Summary

## Source data

Source Data Fig. 1 Statistical source data.

Source Data Fig. 2 Statistical source data.

Source Data Fig. 3 Statistical source data.

Source Data Fig. 6 Statistical source data.

Source Data Extended Data Fig. 2 Statistical source data.

## Data Availability

The data used herein are publicly available in the Analysis and Supplementary Information. Source data are provided with this paper. All other data that support the findings of this study are available from the corresponding authors upon reasonable request.

## Code availability

The dominance analysis package used in this study is developed based on the R programming environment. Basic codes and usage instructions are available at https://www.rdocumentation.org/packages/dominanceanalysis/versions/2.0.0. All codes in this study are available in Supplementary Information.
