# Supplementary material for: Crop rotation increases Tibetan barley yield and soil quality on the Tibetan Plateau
Source: Nat Food. 2025 Jan 28;6(2):151–60. doi: 10.1038/s43016-024-01094-8 (PMC11850288; doi:10.1038/s43016-024-01094-8)
Supplement: Supplementary file 1 — Supplementary Tables 1–6, Fig. 2 code, Fig. 4 code, Fig. 5 code, Fig. 6 code, Extended Data Fig. 1 code and Extended Data Fig. 2 code. [file 43016_2024_1094_MOESM1_ESM.pdf]

nature food

Supplementary information https://doi.org/10.1038/s43016-024-01094-8

Crop rotation increases Tibetan barley yield and soil quality on the Tibetan Plateau

In the format provided by the authors and unedited

Supplementary Information for "*Crop rotation increases Tibetan barley yield and soil quality on the Tibetan Plateau*" by Hui Wu et al

Contents

Supplementary Table 1-5

Supplementary Table 1

Supplementary Table 2

Supplementary Table 3

Supplementary Table 4

Supplementary Table 5

Supplementary Table 6

R language code for figures

Figure 2 code

Figure 4 code

Figure 5 code

Figure 6 code

Extended Data Fig 1 code

Extended Data Fig 2 code

Supplementary Table 1

Table S1. Tibetan barley yield at the sample sites.

	Treatment	Sampling points	Yield(kg/ha)
CC	3(Continuous Cropping)	29°53'14.2"N 91°05'45.1"E	3976
	3(Continuous Cropping)	29°19'20.3"N 88°50'22.0"E	4034
	3(Continuous Cropping)	29°48'00.7"N 93°57'56.6"E	4156
	4(Continuous Cropping)	29°17'35.1"N 91°02'09.1"E	4098
	4(Continuous Cropping)	30°55'39.8"N 97°21'40.9"E	4117
	4(Continuous Cropping)	29°15'23.2"N 87°43'46.7"E	3916
	4(Continuous Cropping)	31°32'31.9"N 92°00'41.6"E	3681
	5(Continuous Cropping)	31°38'42.3"N 96°39'52.5"E	3853
	7(Continuous Cropping)	29°19'58.0"N 88°46'53.6"E	3752
	8(Continuous Cropping)	29°51'15.0"N 91°22'36.7"E	3579
	8(Continuous Cropping)	29°12'25.9"N 91°24'56.8"E	3429
	8(Continuous Cropping)	29°22'42.0"N 94°24'37.2"E	3623
	9(Continuous Cropping)	30°53'45.6"N 97°21'10.1"E	3433
	12(Continuous Cropping)	29°17'34.4"N 91°02'58.0"E	3294
	12(Continuous Cropping)	31°27'45.4"N 92°01'00.3"E	3175
	13(Continuous Cropping)	29°47'31.8"N 93°58'18.5"E	3323
	16(Continuous Cropping)	29°19'58.0"N 88°46'53.6"E	3087
	20(Continuous Cropping)	29°45'05.2"N 91°25'26.0"E	2957
	34(Continuous Cropping)	29°52'32.2"N 91°20'44.4"E	2674
CW	3(Rotating wheat)	29°18'55.6"N 88°38'33.2"E	4353
	3(Rotating wheat)	29°52'31.9"N 91°07'35.3"E	4212
	3(Rotating wheat)	30°53'26.8"N 97°20'50.5"E	4121
	4(Rotating wheat)	29°15'04.5"N 90°55'25.2"E	4328
	5(Rotating wheat)	29°43'13.6"N 94°04'35.5"E	4110
	6(Rotating wheat)	29°15'51.5"N 88°37'08.5"E	3755
	8(Rotating wheat)	31°38'41.9"N 96°39'45.7"E	3973
	10(Rotating wheat)	29°14'24.8"N 90°55'53.5"E	3874
	12(Rotating wheat)	29°29'12.7"N 94°36'31.6"E	3752
	13(Rotating wheat)	29°06'52.6"N 88°54'27.4"E	3592
	15(Rotating wheat)	29°15'06.0"N 87°39'09.2"E	3464
CR	2(Rotating oilseed rape)	29°52'19.9"N 91°19'45.3"E	4162
	3(Rotating oilseed rape)	29°53'22.5"N 91°19'37.3"E	4258
	3(Rotating oilseed rape)	29°10'26.6"N 90°56'14.1"E	4521
	4(Rotating oilseed rape)	29°27'46.8"N 94°38'50.5"E	4393
	5(Rotating oilseed rape)	29°13'24.1"N 87°41'26.7"E	4102
	5(Rotating oilseed rape)	31°39'37.5"N 96°39'30.4"E	4234
	7(Rotating oilseed rape)	29°11'00.5"N 90°55'43.7"E	3994
	8(Rotating oilseed rape)	29°03'42.4"N 88°53'47.4"E	3899
	8(Rotating oilseed rape)	29°29'01.9"N 94°45'35.0"E	3972

Supplementary Table 2

Table S2. Tibetan Barley fields soil properties at the sample sites.

Treatment1	Treatment2	Years	SOM(g/kg)	MBC(mg/kg)	TN(g/kg)	NH_4^+-N(mg/kg)	NO_3^--N(mg/kg)	MBN(mg/kg)	TP(g/kg)	TK(g/kg)	pH
CC	CC5	3	22.966	63.750	1.215	3.842	25.571	15.050	0.590	11.931	7.749
			22.055	73.620	1.119	4.697	26.731	15.700	0.580	11.251	7.749
			22.144	68.550	1.212	5.211	27.771	13.950	0.570	10.940	7.745
		3	24.321	75.780	1.242	4.553	27.781	14.460	0.640	12.070	7.977
			22.955	69.730	1.229	4.193	24.606	14.490	0.530	13.050	7.911
			23.144	78.760	1.188	4.532	25.404	16.190	0.520	12.310	7.774
		3	23.539	74.530	1.286	4.279	27.445	16.010	0.560	10.540	7.679
			25.354	79.310	1.201	4.412	27.622	17.200	0.550	10.730	7.699
			24.232	73.320	1.333	4.547	27.615	15.520	0.590	10.520	7.613
		4	24.776	73.790	1.251	4.215	27.295	15.310	0.560	10.383	7.606
			23.75	75.920	1.182	4.130	26.774	15.820	0.530	9.979	7.612
			23.593	75.860	1.196	4.013	27.622	15.520	0.540	10.720	7.771
		4	24.961	78.960	1.346	3.736	28.581	16.000	0.540	9.990	7.599
			24.512	72.510	1.244	3.621	28.622	15.810	0.560	9.950	7.579
			24.973	77.510	1.339	3.756	28.779	15.350	0.520	9.970	7.586
		4	22.835	68.430	1.093	4.072	24.151	14.940	0.530	14.759	7.665
			22.024	76.150	1.225	3.966	24.517	15.240	0.560	13.344	7.656
			22.568	67.130	1.099	4.071	23.166	15.450	0.550	11.363	7.660
		4	21.499	69.320	1.175	3.534	23.981	14.000	0.480	10.472	7.854
			20.966	77.510	1.092	3.612	22.162	13.550	0.490	11.240	7.843
			21.711	65.020	1.185	3.512	23.862	14.250	0.460	10.124	7.836
		5	22.618	69.320	1.138	3.851	25.267	14.510	0.530	8.311	7.787
			22.015	66.210	1.225	3.751	24.651	15.060	0.520	8.239	7.785
			21.287	72.510	1.185	3.892	25.971	15.020	0.510	10.590	7.778
	CC10	7	23.018	77.410	1.231	3.638	26.462	16.470	0.563	12.120	7.859
			23.877	63.470	1.204	3.918	30.990	16.410	0.518	11.410	7.974
			24.877	64.620	1.224	3.502	30.191	15.570	0.462	11.750	7.958
		8	21.055	61.180	1.135	3.926	23.922	12.380	0.510	8.502	7.943
			20.51	72.220	1.090	3.980	24.795	13.370	0.555	9.830	8.051
			21.244	65.920	1.151	3.761	23.429	14.730	0.537	9.241	8.088
		8	21.024	59.550	1.104	3.698	24.795	14.510	0.520	10.888	8.088
			21.071	65.240	1.048	3.662	22.938	14.520	0.540	10.792	8.179
			20.214	61.410	1.123	3.653	24.633	14.140	0.530	10.616	8.101
		8	22.314	65.310	1.105	3.976	27.436	15.220	0.570	10.751	8.148
			22.931	62.330	1.117	3.885	25.833	16.200	0.520	10.820	8.088
			21.499	65.210	1.123	3.574	29.709	15.850	0.550	10.215	8.115

		9	21.673	61.250	1.092	3.554	24.094	14.550	0.490	11.010	8.072
			20.664	62.490	1.067	3.675	22.074	13.790	0.500	11.755	8.079
			21.518	64.830	1.084	3.464	24.644	12.920	0.510	11.581	8.107
	CC10+	12	19.271	64.510	1.129	3.256	23.937	15.080	0.520	10.134	8.216
			19.375	60.030	1.174	3.353	24.415	15.880	0.530	10.013	8.212
			19.671	67.310	1.157	3.277	23.735	15.440	0.510	10.903	8.273
		12	19.166	65.870	1.083	3.279	23.636	15.720	0.510	10.751	8.228
			19.622	62.310	1.075	3.261	24.024	15.770	0.560	10.851	8.389
			18.568	61.180	1.063	3.243	24.562	15.930	0.520	10.951	8.282
		13	19.569	66.730	1.112	3.532	24.214	16.320	0.520	9.921	8.256
			19.863	67.670	1.157	3.437	25.352	16.020	0.510	9.825	8.176
			19.549	73.830	1.146	3.411	24.462	17.000	0.540	10.086	8.170
		16	18.682	62.880	1.068	3.310	24.661	17.260	0.440	11.936	8.275
			18.693	60.450	1.055	3.075	25.012	15.520	0.480	11.701	8.279
			18.37	65.540	1.061	3.176	24.582	15.330	0.440	11.783	8.268
		20	18.457	63.540	1.075	3.395	23.510	14.940	0.460	10.632	8.318
			18.669	59.500	1.068	3.312	23.622	15.230	0.470	10.823	8.282
			18.479	57.570	0.989	3.254	23.821	13.980	0.480	10.890	8.328
		34	18.077	60.440	0.983	3.277	22.721	15.820	0.520	11.284	8.385
			18.438	60.980	0.966	3.212	21.925	12.640	0.510	11.252	8.339
			18.458	58.720	0.968	3.226	22.488	14.510	0.510	11.193	8.397

Supplementary Table 3

Table S3. *P* value of soil properties in Tibetan barley fields.

p_value	SOM	MBC	TN	NH_4^+-N	NO_3^--N	MBN	TP	TK	pH
SOM	0	4.64E-29	1.21E-26	4.45E-36	5.38E-27	2.63E-18	1.16E-22	0.396414	5.32E-47
MBC	4.64E-29	0	5.79E-14	1.29E-27	3.72E-16	5.11E-23	1.10E-19	0.407739	6.27E-26
TN	1.21E-26	5.79E-14	0	4.01E-18	1.59E-18	9.14E-10	1.39E-12	0.650568	1.09E-24
NH_4^+-N	4.45E-36	1.29E-27	4.01E-18	0	1.12E-16	1.48E-15	8.27E-23	0.241954	3.37E-34
NO_3^--N	5.38E-27	3.72E-16	1.59E-18	1.12E-16	0	5.21E-16	1.35E-15	0.229113	2.71E-22
MBN	2.63E-18	5.11E-23	9.14E-10	1.48E-15	5.21E-16	0	1.20E-11	0.064357	1.27E-13
TP	1.16E-22	1.10E-19	1.39E-12	8.27E-23	1.35E-15	1.20E-11	0	0.120722	1.42E-22
TK	0.396414	0.407739	0.650568	0.241954	0.229113	0.064357	0.120722	0	0.948838
pH	5.32E-47	6.27E-26	1.09E-24	3.37E-34	2.71E-22	1.27E-13	1.42E-22	0.948838	0

Supplementary Table 4

Table S4. Soil quality index of Tibetan barley continuous cropping and rotating cropping fields.

	SOM	MBC	TN	NH_4^+-N	NO_3^--N	MBN	TP	TK	pH	SQI
CC5	1.000	1.000	1.000	1.000	1.000	1.000	1.000	1.000	1.000	1.000
CC10	0.944	0.892	0.932	0.912	0.987	0.969	0.968	0.982	1.042	0.931
CC10+	0.819	0.871	0.889	0.807	0.917	1.019	0.925	0.989	1.071	0.872
CW5	1.138	1.202	1.029	1.248	1.076	1.326	1.100	1.025	0.963	1.222
CW10	1.036	1.179	1.044	1.108	1.054	1.342	1.051	0.977	0.999	1.152
CW10+	0.977	1.144	0.914	0.969	1.025	1.327	1.012	1.094	1.038	1.108
CR5	1.193	1.227	1.053	1.274	1.134	1.325	1.166	1.010	0.932	1.267
CR10	1.106	1.188	1.033	1.148	1.073	1.480	1.085	0.990	0.952	1.205

Supplementary Table 5

Table S5. Structural Equation Modeling regressions of Tibetan barley continuous cropping and rotation.

		Estimate	Std.Err	z-value	P(>|z|)	Std.lv	Std.all
Yield	SOM	48.129	6.324	7.610	0.000	48.129	0.343
	MBC	0.333	1.425	0.234	0.815	0.333	0.008
	TN	615.084	113.216	5.433	0.000	615.084	0.149
	NH4	-2.382	17.556	-0.136	0.892	-2.382	-0.004
	pH	248.619	63.586	3.910	0.000	248.619	0.231
	CR	-227.177	18.764	-12.107	0.000	-227.177	-0.334
	CC	-203.505	35.486	-5.735	0.000	-203.505	-0.552
	CW	-148.160	22.076	-6.711	0.000	-148.160	-0.335
SQI	SOM	0.015	0.002	7.177	0.000	0.015	0.350
	MBC	0.004	0.000	9.487	0.000	0.004	0.340
	TN	0.147	0.042	3.503	0.000	0.147	0.115
	NH4	0.043	0.006	6.741	0.000	0.043	0.256
	pH	0.019	0.017	1.135	0.256	0.019	0.057
	CR	-0.013	0.015	-3.174	0.195	-0.006	-0.039
	CC	-0.088	0.008	-11.232	0.000	-0.088	-0.876
	CW	-0.017	0.009	-1.460	0.001	-0.013	-0.174
SOM	CR	-0.298	0.267	-1.115	0.265	-0.298	-0.062
	C	-2.748	0.156	-17.590	0.000	-2.748	-0.970
	C	-1.382	0.178	-7.783	0.000	-1.382	-0.438
MBC	CR	3.012	1.098	2.742	0.066	3.012	0.164
	CC	-7.611	0.693	-10.984	0.000	-7.611	-0.812
	CW	0.134	0.788	0.170	0.865	0.134	0.012
TN	CR	-0.022	0.015	-1.464	0.143	-0.022	-0.133
	CC	-0.077	0.009	-8.778	0.000	-0.077	-0.858
	CW	-0.048	0.010	-4.866	0.000	-0.048	-0.450
NH_4^+-N	CR	-0.147	0.096	-1.530	0.126	-0.147	-0.117
	CC	-0.653	0.056	-11.610	0.000	-0.653	-0.960
	CW	-0.325	0.064	-5.085	0.000	-0.325	-0.398
pH	CR	0.008	0.027	0.282	0.778	0.008	0.012
	CC	-0.359	0.016	-23.103	0.000	-0.359	-0.970
	CW	-0.223	0.018	-12.645	0.000	-0.223	-0.542

Supplementary Table 6

Table S6. Duncan multiple comparative analysis of soil properties in Tibetan barley fields.

treat	mean	label	sd	se	index
CC5	3978.875	b	158.6627	56.09572	Yield
CW5	4224.8	a	113.1402	50.59783	Yield
CR5	4278.333	a	154.2993	62.99242	Yield
CC10	3563.2	b	136.4119	61.00525	Yield
CW10	3867.333	a	109.1528	63.0194	Yield
CR10	3955	a	49.72927	28.71121	Yield
CC10+	3085	b	242.3774	98.95016	Yield
CW10+	3602.667	a	144.296	83.30933	Yield
CC5	23.11658	c	1.281635	0.261613	SOM
CW5	26.3106	b	0.913352	0.235826	SOM
CR5	27.57456	a	1.455517	0.343069	SOM
CC10	21.8326	c	1.321439	0.341194	SOM
CW10	23.95	b	1.293588	0.431196	SOM
CR10	25.57133	a	0.341852	0.113951	SOM
CC10+	18.94317	a	0.558718	0.131691	SOM
CW10+	22.58133	a	0.744602	0.248201	SOM
CC5	72.64583	b	4.628564	0.944802	MBC
CW5	87.314	a	3.600115	0.929546	MBC
CR5	89.17	a	5.037745	1.187408	MBC
CC10	64.82933	b	4.58205	1.18308	MBC
CW10	85.63111	a	5.883087	1.961029	MBC
CR10	86.31444	a	5.483735	1.827912	MBC
CC10+	63.28111	a	4.038332	0.951844	MBC
CW10+	83.10111	a	4.110898	1.370299	MBC
CC5	1.208333	b	0.071656	0.014627	TN
CW5	1.243333	ab	0.087646	0.02263	TN
CR5	1.271833	a	0.070665	0.016656	TN
CC10	1.126533	b	0.054915	0.014179	TN
CW10	1.261889	a	0.10382	0.034607	TN
CR10	1.248222	a	0.044525	0.014842	TN
CC10+	1.073833	a	0.065369	0.015408	TN
CW10+	1.105	a	0.05194	0.017313	TN
CC5	4.08325	b	0.41875	0.085477	NH_4^+-N
CW5	5.095267	a	0.498949	0.128828	NH_4^+-N
CR5	5.200944	a	0.543705	0.128152	NH_4^+-N
CC10	3.7244	b	0.173701	0.044849	NH_4^+-N

CW10	4.523556	a	0.620261	0.206754	NH_4^+-N
CR10	4.685889	a	0.059675	0.019892	NH_4^+-N
CC10+	3.293667	a	0.104547	0.024642	NH_4^+-N
CW10+	3.956111	a	0.176347	0.058782	NH_4^+-N
CC5	26.08113	c	1.888707	0.385531	NO_3^--N
CW5	28.06053	b	2.358944	0.609077	NO_3^--N
CR5	29.5715	a	1.239688	0.292197	NO_3^--N
CC10	25.72967	b	2.717539	0.701666	NO_3^--N
CW10	27.47789	ab	1.091649	0.363883	NO_3^--N
CR10	27.98789	a	0.999457	0.333152	NO_3^--N
CC10+	23.92661	a	0.878768	0.207128	NO_3^--N
CW10+	26.728	a	1.548769	0.516256	NO_3^--N
CC5	15.18333	b	0.830844	0.169595	MBN
CW5	20.14	a	2.168281	0.559848	MBN
CR5	20.11556	a	1.862693	0.439041	MBN
CC10	14.70867	c	1.259506	0.325203	MBN
CW10	20.37333	b	2.98177	0.993923	MBN
CR10	22.57889	a	0.547276	0.182425	MBN
CC10+	15.46611	a	1.054085	0.24845	MBN
CW10+	20.15111	a	1.953212	0.651071	MBN
CC5	0.542083	c	0.038559	0.007871	TP
CW5	0.5962	b	0.028773	0.007429	TP
CR5	0.632333	a	0.035843	0.008448	TP
CC10	0.525	b	0.028978	0.007482	TP
CW10	0.569889	a	0.063456	0.021152	TP
CR10	0.588	a	0.023098	0.007699	TP
CC10+	0.501667	a	0.033122	0.007807	TP
CW10+	0.548667	a	0.047964	0.015988	TP
CC5	10.949	a	1.459843	0.297989	TK
CW5	11.2228	a	1.019429	0.263215	TK
CR5	11.05306	a	0.610455	0.143886	TK
CC10	10.75207	a	0.985158	0.254367	TK
CW10	10.694	a	0.54841	0.182803	TK
CR10	10.83967	a	0.223444	0.074481	TK
CC10+	10.82939	a	0.642481	0.151434	TK
CW10+	11.97422	a	0.961056	0.320352	TK
CC5	7.729708	a	0.107974	0.02204	pH
CW5	7.4452	b	0.13163	0.033987	pH
CR5	7.207333	c	0.137988	0.032524	pH
CC10	8.056667	a	0.085974	0.022198	pH
CW10	7.719333	b	0.13747	0.045823	pH
CR10	7.36	c	0.058654	0.019551	pH
CC10+	8.281833	a	0.068129	0.016058	pH

CW10+	8.02	a	0.066695	0.022232	pH
CC5	4.08325	a	0.41875	0.085477	NH_4^+-N
CC10	3.7244	b	0.173701	0.044849	NH_4^+-N
CC10+	3.293667	c	0.104547	0.024642	NH_4^+-N
CC5	26.08113	a	1.888707	0.385531	NO_3^--N
CC10	25.72967	a	2.717539	0.701666	NO_3^--N
CC10+	23.92661	b	0.878768	0.207128	NO_3^--N
CC5	23.11658	a	1.281635	0.261613	SOM
CC10	21.8326	b	1.321439	0.341194	SOM
CC10+	18.94317	c	0.558718	0.131691	SOM
CC5	72.64583	a	4.628564	0.944802	MBC
CC10	64.82933	b	4.58205	1.18308	MBC
CC10+	63.28111	b	4.038332	0.951844	MBC
CC5	1.208333	a	0.071656	0.014627	TN
CC10	1.126533	b	0.054915	0.014179	TN
CC10+	1.073833	c	0.065369	0.015408	TN
CC5	15.18333	ab	0.830844	0.169595	MBN
CC10	14.70867	b	1.259506	0.325203	MBN
CC10+	15.46611	a	1.054085	0.24845	MBN
CC5	0.542083	a	0.038559	0.007871	TP
CC10	0.525	a	0.028978	0.007482	TP
CC10+	0.501667	b	0.033122	0.007807	TP
CC5	10.949	a	1.459843	0.297989	TK
CC10	10.75207	a	0.985158	0.254367	TK
CC10+	10.82939	a	0.642481	0.151434	TK
CC5	7.729708	c	0.107974	0.02204	pH
CC10	8.056667	b	0.085974	0.022198	pH
CC10+	8.281833	a	0.068129	0.016058	pH

Figure 2 code

```
rm(list=ls())
library(dplyr)
library(ggplot2)
library(agricolae)
library(ggpubr)
rm(list=ls())
mydata=data.frame(readxl::read_excel('715.xlsx', sheet = 2),check.names = F)
mydata[,2:ncol(mydata)] = as.numeric(unlist(mydata[,2:ncol(mydata)]))
class(mydata[,2])
barplot_2treat_duncan_test = function(n){
  data = mydata[,c(1,(n+1),ncol(mydata))]
  colnames(data)[1] = 'trt'#treatment
  data = split(data, data$trt)

  for (i in 1:length(data)) {
    aov = aov(data[[i]][,2] ~ treat, data = data[[i]])
    duncan = agricolae::duncan.test(aov, trt = 'treat')

    duncan$means$se = NA
    for (m in 1:nrow(duncan$means)) {
      duncan$means$se[m] = duncan$means$std[m] / sqrt(duncan$means$r[m])
    }

    total = merge(duncan$groups, duncan$means[,c('std','se')], by = 'row.names')
    total
    colnames(total) = c('treat', 'mean', 'label', 'sd', 'se')
    total$treat = factor(total$treat, levels = unique(mydata[,ncol(mydata)]))
    total = total %>% arrange(treat)

    total$'trt' = names(data)[i]
    data[[i]] = total
  }
  data = data.table::rbindlist(data)

  data$trt = factor(data$trt, levels = unique(mydata[,1]))
  data$treat = factor(data$treat, levels = c(2,1,3))
  data = data %>% arrange(trt)

  p = ggplot(data, aes(x = trt, y = mean, fill=treat)) +

    geom_bar( stat = "identity", position="dodge") +
```

```
    geom_errorbar(aes(ymin = mean - se, ymax = mean + se), position =
position_dodge(0.9), width=0.7) +

    geom_text(aes(y = mean + se, label = label), vjust = -0.5,
position=position_dodge(0.9), check_overlap = T) +
    # geom_text(aes(label = label, vjust = -0.5, hjust = 'center'), size=15, check_overlap =
T) +

    labs(x = colnames(data)[1], y = colnames(mydata)[n+1]) +
    scale_fill_brewer(palette = 'Set2') +
    theme(panel.background = element_blank(),
          axis.line = element_line(colour = "black"),
          # axis.ticks = element_line(size = 2),axis.ticks.length=unit (0.15, "cm"),
          axis.text = element_text(colour = 'black'),
          axis.text.y = element_text(family=windowsFont("Arial")),
          axis.text.x = element_text(family=windowsFont("Arial"), angle = 45, hjust = 1),
          axis.title.x = element_blank(),
          axis.title.y = element_text(family=windowsFont("Arial")),
          legend.title = element_blank(),
          #legend.position = "none",
          panel.border = element_rect(fill=NA,color="black", linetype="solid"),
          plot.margin=unit(rep(1,4),'lines')) +
    coord_cartesian(ylim = c(0, (max(data$mean + data$se))*1.2))
  p
}

mydf = data.frame()
for (i in 1:9) {
  mydf[i,1] = paste0('barplot_2treat_duncan_test(',i,')')
}
print(paste(mydf[,1], collapse = ','))

p4 = barplot_2treat_duncan_test(4)+
  ylab(expression(NH[4]^{"+"}-N(mg/kg)))
p5 = barplot_2treat_duncan_test(5)+
  ylab(expression(NO[3]^{"-"}-N(mg/kg)))

png(file="plot2.png",width = 2600, height = 2200, res = 300)
ggpubr::ggarrange(barplot_2treat_duncan_test(1),barplot_2treat_duncan_test(2),barplot_
2treat_duncan_test(3),p4,p5,barplot_2treat_duncan_test(6),barplot_2treat_duncan_test(
7),barplot_2treat_duncan_test(8),barplot_2treat_duncan_test(9),
                  labels = 'AUTO',common.legend = T, legend = "top")
dev.off()
```

Figure 4 code

```
rm(list=ls())
library("randomForest")
n<-length(names(train_data))
rate=1
for(i in 1:(n-1)){
  set.seed(1234)
  rf_train<-
randomForest(as.factor(train_data$IS_LIUSHI)~.,data=train_data,mtry=i,ntree=1000)
  rate[i]<-mean(rf_train$err.rate)
  print(rf_train)
}
rate
plot(rate)

set.seed(100)
rf_train<-
randomForest(as.factor(train_data$IS_LIUSHI)~.,data=train_data,mtry=12,ntree=1000)
plot(rf_train)
legend(800,0.02,"IS_LIUSHI=0",cex=0.9,bty="n")
legend(800,0.0245,"total",cex=0.09,bty="n")

set.seed(100)
rf_train<-
randomForest(as.factor(train_data$IS_LIUSHI)~.,data=train_data,mtry=12,ntree=400,imp
ortance=TRUE,proximity=TRUE)

importance<-importance(rf_train)
write.csv(importance,file="E:/model/importance.csv",row.names=T,quote=F)
barplot(rf_train$importance[,1],main="Variable importance measure indicator histogram
")
box()

importance(rf_train,type=1)
varImpPlot(x=rf_train,sort=TRUE,n.var=nrow(rf_train$importance),main="Variable
importance measure indicator histogram
")

print(rf_train)
hist(treesize(rf_train))
max(treesize(rf_train));min(treesize(rf_train))
MDSplot(rf_train,train_data$IS_OFF_USER,palette=rep(1,2),pch=as.numeric(train_data$
```

```
IS_LIUSHI))

pred<-predict(rf_train,newdata=test_data)
pred_out_1<-predict(object=rf_train,newdata=test_data,type="prob")
table <- table(pred,test_data$IS_LIUSHI)
sum(diag(table))/sum(table)
plot(margin(rf_train,test_data$IS_LIUSHI),main=Variable importance measure indicator
histogram
)
```

Figure 5 code

```
rm(list=ls())
library(vegan)
library(ggrepel)
library(ggplot2)
library(ggpubr)
windowsFonts(A=windowsFont("Arial"),
             B=windowsFont("Arial"))

sampledata <- read.csv(file.choose(), head = TRUE, row.names=1)
env <- read.csv(file.choose(), header=TRUE, row.names=1)
group <-   read.csv(file.choose(), header = FALSE,
                    colClasses=c("character"))
sampledata <- t(sampledata)
sampledata <- decostand(sampledata,method = "hellinger")
group <- as.list(group)
dca <- decorana(veg = sampledata)
dca1 <- max(dca$rproj[,1])
dca2 <- max(dca$rproj[,2])
dca3 <- max(dca$rproj[,3])
dca4 <- max(dca$rproj[,4])
GL <- data.frame(DCA1 = c(dca1), DCA2 = c(dca2), DCA3 = c(dca3), DCA4 = c(dca4))
GL
> GL
rownames(GL) <- c("Gradient length")
write.csv(GL, file = "dca.csv")
rda <- rda(sampledata, env, scale = TRUE)
rdascore <- scores(rda)
rdascore$sites
rda$CCA$biplot
rdascore$species
write.csv(rdascore$sites,file="rda.sample.csv")
write.csv(rda$CCA$biplot,file="rda.env.csv")
write.csv(rdascore$species,file="rda.species.csv")

RDAE <- as.data.frame(rda$CCA$biplot[,1:2])
RDAS1 <- rdascore$sites[,1]*0.2
RDAS2 <- rdascore$sites[,2]*0.2

plotdata <- data.frame(rownames(rdascore$sites), RDAS1, RDAS2, group$V2)
colnames(plotdata) <- c("sample","RDAS1","RDAS2","group")
```

```
rda1 <- round(rda$CCA$eig[1]/sum(rda$CCA$eig)*100,2)
rda2 <- round(rda$CCA$eig[2]/sum(rda$CCA$eig)*100,2)

PP <-ggplot(plotdata, aes(RDAS1, RDAS2)) +
  geom_point(aes(fill = group, color = group),size = 5) +
  scale_fill_manual(values = col)+
  stat_chull(geom = "polygon", aes(group = group, color = group, fill = group), alpha =
0.1)+
  xlab(paste("RDA1 ( ",rda1,"%"," )", sep = "")) +
  ylab(paste("RDA2 ( ",rda2,"%"," )", sep = "")) +
  geom_segment(data = RDAE, aes(x = 0, y = 0, xend = RDAE[,1], yend = RDAE[,2]),
               colour = "black", size = 0.8,
               arrow = arrow(angle = 30, length = unit(0.4, "cm"))) +
  geom_text_repel(data = RDAE, segment.colour = "black",
                  aes(x = RDAE[,1], y = RDAE[,2], label = rownames(RDAE)),size=8) +
  geom_vline(aes(xintercept = 0), linetype = "dotted") +
  geom_hline(aes(yintercept = 0), linetype = "dotted") +
  theme(panel.background = element_rect(fill = "white", colour = "black"),
        panel.grid = element_blank(),
        axis.title = element_text(color = "black", size = 18),
        axis.ticks.length = unit(0.4,"lines"),
        axis.ticks = element_line(color = "black"),
        axis.line = element_line(colour = "black"),
        axis.title.x = element_text(colour = "black", size = 18),
        axis.title.y = element_text(colour="black", size = 18),
        axis.text = element_text(colour = "black", size = 18),
        legend.title = element_blank(),
        legend.text = element_text(size = 18), legend.key = element_blank(),
        plot.title = element_text(size = 22, colour = "black",
                                  face = "bold", hjust = 0.5)) +
  theme(text=element_text(family="A",size=20))
PP
library(eoffice)
topptx(filename="RDA.pptx",height = 6,width = 8)
envfit <- envfit(rda, env, permutations   = 999)
r <- as.matrix(envfit$vectors$r)
p <- as.matrix(envfit$vectors$pvals)
env.p <- cbind(r,p)
colnames(env.p) <- c("r2","p-value")
KK <- as.data.frame(env.p)
KK
write.csv(as.data.frame(env.p),file="rdaenvfit.csv")
```

Figure 6

```
library(lavaan)
library(haven)
library(Hmisc)
library(semPlot)
rm(list = ls())
ls()
setwd("D:/11/CC")
data=read.table ("SEM.txt",sep="\t", h=TRUE)
attach(data)
data
d<-data[,c(2:15)]
d
df=scale(d,center=TRUE,scale=TRUE)
df

model <- '
          Yield ~ SOM + MBC + TN + NH4 + pH
          Yield ~ CR + CC + CW
          SQI ~ SOM + MBC + TN + NH4 + pH
          SQI ~ CR + CC + CW
          SOM ~ CR + CC + CW
          MBC ~ CR + CC + CW
          TN ~ CR + CC + CW
          NH4 ~ CR + CC + CW
          pH ~ CR + CC + CW
          '
fit_model<-sem(model,data=d)
resid(fit_model, type = "cor")

summary(fit_model,standardized=T)
summary(fit_model, fit.measures=T)
modindices(fit_model,sort.=TRUE)
inspect(fit_model,what="std")
inspect(fit_model,"r2")
fitmeasures(fit_model,c("chisq", "df", "gfi", "rmsea", "srmr"))
fitMeasures(fit_model,c("chisq","df","pvalue","cfi","nfi","ifi","rmsea","EVCI"))

semPaths(fit_model,"std",edge.label.cex=0.8,
           fade=FALSE, layout = "spring",
           optimizeLatRes = FALSE, residuals = FALSE)
```

Extended Data Fig 1 code

```
rm(list=ls())
library(dplyr)
library(ggplot2)
library(agricolae)
library(ggpubr)

mydata=data.frame(readxl::read_excel('715.xlsx', sheet = 1),check.names = F)
mydata[,2:ncol(mydata)] = as.numeric(unlist(mydata[,2:ncol(mydata)]))
class(mydata[,2])

box_plot_duncan_test = function(n){
  {
  data = mydata[,c(1,(n+1))]
  colnames(data)[1] = 'trt'

  aov = aov(data[,2] ~ trt, data = data)
  summary(aov)

  duncan = agricolae::duncan.test(aov, trt = 'trt')
  duncan$groups
  duncan$means$std

  duncan$means$se = NA
  for (m in 1:nrow(duncan$means)) {
    duncan$means$se[m] = duncan$means$std[m] / sqrt(duncan$means$r[m])
  }

  total = merge(duncan$groups, duncan$means[,c('std','se')], by = 'row.names')
  total
  colnames(total) = c('trt', 'mean', 'label', 'sd', 'se')
  total$trt = factor(total$trt, levels = unique(mydata[,1]))
  total = total %>% arrange(trt)
  }

  p = aggregate(data[,2], by = list(trt = data$trt), boxplot) %>% as.matrix() %>%
as.data.frame()
  p$trt = factor(p$trt, levels = unique(data$trt))
  p = (p %>% arrange(trt))$x.stats %>% unlist()
  total$'boxplot_max' = p[seq(from = 0, to = length(p), by = 5) %>% .[2:length(.)]]

  data$trt = factor(data$trt, levels = unique(data$trt))
```

```
  data = merge(data, total) %>% arrange(trt)

  p = ggplot(data, aes(x = trt, y = data[,2], fill = trt)) +
    geom_boxplot(outlier.shape = NA) +
    stat_boxplot(geom = "errorbar", width = 0.5) +
    geom_text(aes(y = boxplot_max, label = label, vjust = -0.5),
family=windowsFont("serif"), check_overlap = T) +

    labs(x = colnames(data)[1], y = colnames(mydata)[n+1]) +

    theme(panel.background = element_blank(),
          axis.line = element_line(colour = "black"),
          # axis.ticks = element_line(size = 2),axis.ticks.length=unit (0.15, "cm"),
          axis.text = element_text(colour = 'black'),
          axis.text.y = element_text(family=windowsFont("serif")),
          axis.text.x = element_text(family=windowsFont("serif"), angle = 45, hjust = 1),
          axis.title.x = element_blank(),
          axis.title.y = element_text(family=windowsFont("serif")),
          legend.position = "none",
          panel.border = element_rect(fill=NA,color="black", linetype="solid"),
          plot.margin=unit(rep(1,4),'lines')) +
    coord_cartesian(ylim = c(0, (max(data$boxplot_max))*1.6))
  p
}

mydf = data.frame()
for (i in 1:9) {
  mydf[i,1] = paste0('box_plot_duncan_test(',i,')')
}
print(paste(mydf[,1], collapse = ','))

p4 = box_plot_duncan_test(4)+
  ylab(expression(NH[4]^{"+"}-N(mg/kg)))+
  theme(axis.title.y = element_text(size = 11))
p5 = box_plot_duncan_test(5)+
  ylab(expression(NO[3]^{"-"}-N(mg/kg)))+
  theme(axis.title.y = element_text(size = 11))

png(file="plot1.png",width = 1700, height = 1700, res = 300)
cowplot::plot_grid(box_plot_duncan_test(1),box_plot_duncan_test(2),box_plot_duncan_t
est(3),p4,p5,box_plot_duncan_test(6),box_plot_duncan_test(7),box_plot_duncan_test(8),
box_plot_duncan_test(9),
                   labels = 'AUTO',label_x = -0, nrow = 3)
dev.off()
```

Extended Data Fig 2 code

```
rm(list=ls())

setwd('D:\\Xizangfig\\2418')
df = data.frame(readxl::read_excel('2418.xlsx', sheet = 1),check.names = F)
df = log(df+1)
library(corrgram)
pairs(df)
panel.fill<- function(x, y, digits = 2, prefix = "",col = "red", cex.cor, ...)
{
  par(usr = c(0, 1, 0, 1))
  r <- abs(cor(x, y))
  txt <- format(r, digits = digits)[1]
  col <- colorRampPalette(c("grey",'grey','grey', 'red'))(100)
  rect(0, 0, 1, 1, col = col[ceiling(r * 100)])
  text(0.5, 0.5, txt, cex = 1.5,col = '#77787b', font = 2 )
}

pairs(df,
      lower.panel = panel.fill,
      gap = 0)

panel.point <- function(x, y, ...){
  r <- abs(cor(x, y))
  col <- colorRampPalette(c("grey",'grey','grey', 'red'))(100)
  rect(par("usr")[1], par("usr")[3], par("usr")[2], par("usr")[4],
       col = col[ceiling(r * 100)],lwd = 2)
  plot.xy(xy.coords(x, y), type = "p",
          pch = 20,
          cex = .2,
          ...)
}

pairs(df,
      upper.panel = panel.point,
      lower.panel = panel.fill,
      gap = 0)

text.panel <- function(x, y, txt, cex, ...)
{ text(x, y, txt, cex = cex, font = 2)
  box(lwd = 1)
}
```

```
pairs(df,
      upper.panel = panel.point,
      lower.panel = panel.fill,
      text.panel = text.panel,
      gap = 0)
png(file = "output.png", width = 1700, height = 1700, res = 300)

pairs(df,
      upper.panel = panel.point,
      lower.panel = panel.fill,
      text.panel = text.panel,
      gap = 0)

dev.off()
```
