# Supplementary material for: Crop rotation increases Tibetan barley yield and soil quality on the Tibetan Plateau
Source: Nat Food. 2025 Jan 28;6(2):151–60. doi: 10.1038/s43016-024-01094-8 (PMC11850288; doi:10.1038/s43016-024-01094-8)
Supplement: Supplementary file 2 — Reporting Summary [file 43016_2024_1094_MOESM2_ESM.pdf]

nature portfolio

Corresponding author(s): Xurong Mei
Last updated by author(s): Nov 1, 2024

Reporting Summary

Nature Portfolio wishes to improve the reproducibility of the work that we publish. This form provides structure for consistency and transparency in reporting. For further information on Nature Portfolio policies, see our Editorial Policies and the Editorial Policy Checklist.

Statistics

For all statistical analyses, confirm that the following items are present in the figure legend, table legend, main text, or Methods section.

n/a	Confirmed	
☐	☒	The exact sample size (*n*) for each experimental group/condition, given as a discrete number and unit of measurement
☐	☒	A statement on whether measurements were taken from distinct samples or whether the same sample was measured repeatedly
☐	☒	The statistical test(s) used AND whether they are one- or two-sided<br>*Only common tests should be described solely by name; describe more complex techniques in the Methods section.*
☐	☒	A description of all covariates tested
☐	☒	A description of any assumptions or corrections, such as tests of normality and adjustment for multiple comparisons
☐	☒	A full description of the statistical parameters including central tendency (e.g. means) or other basic estimates (e.g. regression coefficient) AND variation (e.g. standard deviation) or associated estimates of uncertainty (e.g. confidence intervals)
☐	☒	For null hypothesis testing, the test statistic (e.g. *F*, *t*, *r*) with confidence intervals, effect sizes, degrees of freedom and *P* value noted<br>*Give P values as exact values whenever suitable.*
☒	☐	For Bayesian analysis, information on the choice of priors and Markov chain Monte Carlo settings
☐	☒	For hierarchical and complex designs, identification of the appropriate level for tests and full reporting of outcomes
☐	☒	Estimates of effect sizes (e.g. Cohen's *d*, Pearson's *r*), indicating how they were calculated

Our web collection on statistics for biologists contains articles on many of the points above.

Software and code

Policy information about availability of computer code

Data collection	No software is used to collect data
Data analysis	The data analysis was conducted in R (version R4.3.2) with the following packages and versions: Lavaan (0.6), haven (2.5.4), Hmisc (5.1), semPlot (1.1.6), corrgram (1.14), agricolae (1.3), randomForest (4.7), vegan (2.6), ggrepel (0.9.5), ggplot2 (3.5.1), ggpubr (0.6.0).

For manuscripts utilizing custom algorithms or software that are central to the research but not yet described in published literature, software must be made available to editors and reviewers. We strongly encourage code deposition in a community repository (e.g. GitHub). See the Nature Portfolio guidelines for submitting code & software for further information.

Data

Policy information about availability of data

All manuscripts must include a data availability statement. This statement should provide the following information, where applicable:

- Accession codes, unique identifiers, or web links for publicly available datasets
- A description of any restrictions on data availability
- For clinical datasets or third party data, please ensure that the statement adheres to our policy

All data generated in this study are available in the Supplementary Information.

Research involving human participants, their data, or biological material

Policy information about studies with human participants or human data. See also policy information about sex, gender (identity/presentation), and sexual orientation and race, ethnicity and racism.

Reporting on sex and gender	not applicable
Reporting on race, ethnicity, or other socially relevant groupings	not applicable
Population characteristics	not applicable
Recruitment	not applicable
Ethics oversight	not applicable

Note that full information on the approval of the study protocol must also be provided in the manuscript.

Field-specific reporting

Please select the one below that is the best fit for your research. If you are not sure, read the appropriate sections before making your selection.

☐ Life sciences ☐ Behavioural & social sciences ☒ Ecological, evolutionary & environmental sciences

For a reference copy of the document with all sections, see nature.com/documents/nr-reporting-summary-flat.pdf

Ecological, evolutionary & environmental sciences study design

All studies must disclose on these points even when the disclosure is negative.

Study description	We considered the three food crops (Tibetan barley, wheat, rape) to implement rotations and improve crop productivity and soil quality in the Tibetan Plateau. We conducted 39 field experimental sites on the Tibetan Plateau, comparing short-term (≤ 5 years), 5-10 years and long-term (≥ 10 years) continuous cropping and rotation of Tibetan barley with wheat or rape. The objectives of this study were: (1) exploring the effects of type and duration of Tibetan barley rotation on soil quality and yield on the Tibetan Plateau, (2) exploring the driving factors affecting crop yield and soil quality in Tibetan barley rotations, and (3) exploring the suitable rotation planting pattern for Tibetan barley on the Tibetan Plateau.
Research sample	In March 2022, a systematic survey of Tibetan barley fields was carried out in the main production area of Tibetan barley in the Tibetan valley agricultural region. In the valley agricultural region of the Tibetan Plateau, 39 fields of Tibetan barley were selected, including Tibetan barley continuous cropping, Tibetan barley-wheat rotation, Tibetan barley-rape rotation.
Sampling strategy	Soil sampling in these fields was conducted before Tibetan barley planting, from April 5 to April 15, 2022. Three surface composite soil samples (0-20 cm) (made of 5 cores) were taken along the diagonal of each Tibetan barley field. Therefore, we collected a total of 126 samples. Soils were homogenized and sieved through 2 mm, and visible shoot litter and root, as well as stones, were picked out. Then each sample was separated into two parts.
Data collection	Soils were homogenized and sieved through 2 mm, and visible shoot litter and root, as well as stones, were picked out. Then each sample was separated into two parts. One was stored at 4 °C and was used to measure soil microbial biomass carbon (MBC) and microbial biomass nitrogen (MBN) within one week. The other part was air-dried for the determination of soil physicochemical properties. In September 2022, the yield data of Tibetan barley at the sampling points were collected from the farm yield record table.
Timing and spatial scale	In March 2022, a systematic survey of Tibetan barley fields was carried out in the main production area of Tibetan barley in the Tibetan valley agricultural region. In the valley agricultural region of the Tibetan Plateau, 39 fields of Tibetan barley were selected, including Tibetan barley continuous cropping, Tibetan barley-wheat rotation, Tibetan barley-rape rotation (Fig 1A and Supplementary Tables 1).
Data exclusions	No data were excluded from the analyses.
Reproducibility	The results can be reproduced.
Randomization	The spatial analysis section effectively extracted fragmented cropland within the study area, avoiding the need for random sampling.
Blinding	Blinding was not necessary as none of the data used in this study was subjective nor could be influenced by researcher biases.

Did the study involve field work? ☒ Yes ☐ No

Field work, collection and transport

Field conditions	The Tibetan valley agricultural region has a plateau temperate semi-arid monsoon climate. The average annual temperature at all experimental sites is between 6-8 °C, and the average annual precipitation is between 450-550mm.
Location	Tibetan Plateau (Fig 1A and Supplementary Tables 1)
Access & import/export	39 experimental plots in different cropping years were co-managed by the Tibet Academy of Agriculture and Animal Husbandry Sciences and farmers for crop rotation and continuous cropping research.
Disturbance	There was no disturbance.

Reporting for specific materials, systems and methods

We require information from authors about some types of materials, experimental systems and methods used in many studies. Here, indicate whether each material, system or method listed is relevant to your study. If you are not sure if a list item applies to your research, read the appropriate section before selecting a response.

Materials & experimental systems

n/a	Involved in the study
☒	☐ Antibodies
☒	☐ Eukaryotic cell lines
☒	☐ Palaeontology and archaeology
☒	☐ Animals and other organisms
☒	☐ Clinical data
☒	☐ Dual use research of concern
☒	☐ Plants

Methods

n/a	Involved in the study
☒	☐ ChIP-seq
☒	☐ Flow cytometry
☒	☐ MRI-based neuroimaging

Plants

Seed stocks	*Report on the source of all seed stocks or other plant material used. If applicable, state the seed stock centre and catalogue number. If plant specimens were collected from the field, describe the collection location, date and sampling procedures.*
Novel plant genotypes	*Describe the methods by which all novel plant genotypes were produced. This includes those generated by transgenic approaches, gene editing, chemical/radiation-based mutagenesis and hybridization. For transgenic lines, describe the transformation method, the number of independent lines analyzed and the generation upon which experiments were performed. For gene-edited lines, describe the editor used, the endogenous sequence targeted for editing, the targeting guide RNA sequence (if applicable) and how the editor was applied.*
Authentication	*Describe any authentication procedures for each seed stock used or novel genotype generated. Describe any experiments used to assess the effect of a mutation and, where applicable, how potential secondary effects (e.g. second site T-DNA insertions, mosiacism, off-target gene editing) were examined.*
